# Are Psycho-Behavioral Factors Accounting for Longevity?

**DOI:** 10.3389/fpsyg.2019.02516

**Published:** 2019-11-14

**Authors:** Rocío Fernández-Ballesteros, Macarena Sánchez-Izquierdo

**Affiliations:** ^1^Department of Psychobiology and Health, Autonomous University of Madrid, Madrid, Spain; ^2^Department of Psychology, Universidad Pontificia Comillas, Madrid, Spain

**Keywords:** aging, predictors of longevity, psycho-behavioral factors, healthy longevity, successful longevity

## Abstract

The general objective of this article is to critically review the state of the art regarding current factors accounting for aging, longevity and successful longevity. There are two major constructs which most authors are employing to account for longevity: genetic or intrinsic components versus environmental or extrinsic factors. This classification has important flaws: (i) From an epigenetic standpoint, such a polar classification could lead to misconceptions since both factors are interdependent through lifelong interactions. (ii) There are no specifications regarding these “environmental” factors, which include a broad heterogeneity of conditions (physical, economic, social, and cultural aspects as well as behavioral ones such as lifestyle) but do not include personal conditions, such as psychological characteristics. The review of the new paradigm called successful aging yields an important set of psycho-behavioral factors, and although population indexes such as Disability Free Life Expectancy (DFLE) or Healthy Life expectancy (HLE) have been developed, authors do not take into consideration healthy or successful longevity as a potential prolongation of the new paradigm of active or successful aging. There is a broad corpus of research literature supporting the importance of psycho-behavioral (PB) factors intervening in the ways of aging, specifically intelligence and cognitive functioning, positive emotion and control, personality traits, psychosocial, physical conditions, and lifestyles, all of which are highly associated with active aging, health, longevity, and survival. The importance of these factors accounting for longevity, and successful longevity must be taken into consideration as a pending issue in gerontology.

## The Problem: Toward the Aging of Aging

Since the middle of the 19th century, life expectancy has risen rapidly; according to Riley (2005), in the European region in 1850, life expectancy at birth (LE) was 36.3 years, and in 2001 it was 76.8, i.e., twice as high. Experts assume that this phenomenon is the result of a decline in mortality, not only at birth but (from the middle of the 20th century) across all ages.

**FIGURE 1:**
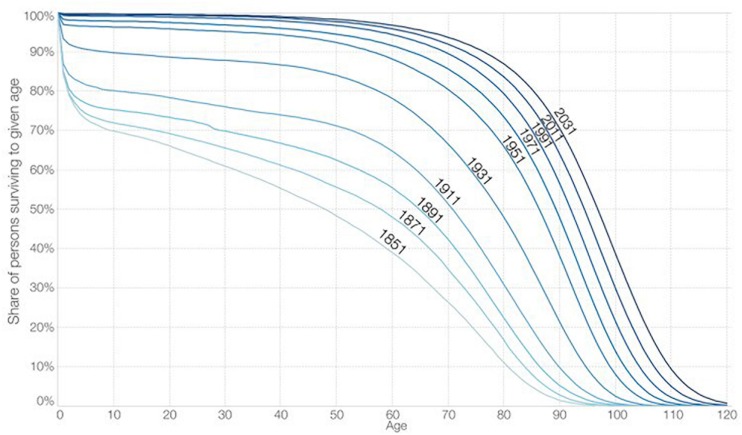
Share of persons surviving to successive ages for persons born 1851 to 2031, England and Wales. Office for National Statistics (ONS). Life expectancy figures are not available for the United Kingdom before 1951; for long historic trends England and Wales data are used. The Interactive data visualization is available at OurWorldinData.org. There you find the raw data and more visualizations on this topic. Licensed under CC-BY-SA by the author Max Roser.

Perhaps the most evident perception of the changes regarding longevity comes from survival curves by Roser (2018), plotting survival curves for individuals born at different points in time and using cohort life tables; this seems to be a good test of Fries’ (see: Fries, 1980; Fries and Crapo, 1981) hypothesis about the *compression* of morbimortality across the *rectangularization* of survival curves; in other words, while fewer than 50% of people born in 1851 lived beyond their 50th birthday, today, more than 95% can expect to live longer than 50 years (see Figure 1).

**FIGURE 2:**
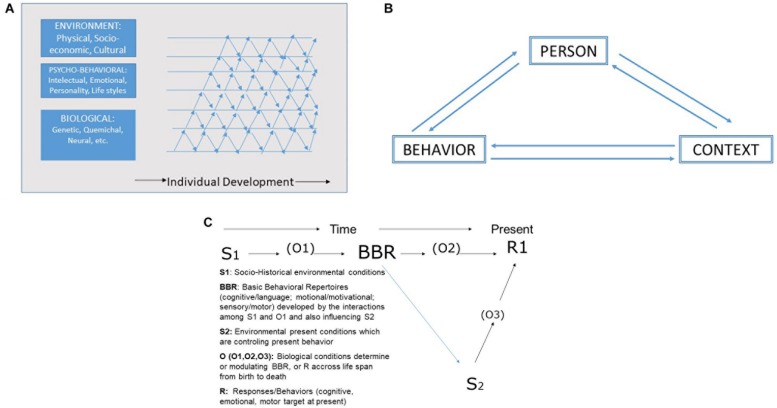
**(A)** The probabilistic-epigenetic framework (modified from Gottlieb, 1991). Reprinted with the permission of the copyright holder. Copyright by American Psychological Association. **(B)** Reciprocal Determinism Theory. The person, his/her behavior and the context, all mutually influence each other (reciprocal determination) (Modified from Bandura, 1978). Reprinted with the permission of the copyright holder. Copyright by American Psychological Association. **(C)** Environment, Organism, Basic Behavioral Repertoires (BBRs) and Behaviors Interactions across Life Cycle (Modified from Fernández-Ballesteros and Staats, 1992). Reprinted with the permission of the copyright holder. Copyright by Advances in Behavior Research an Therapy.

Increasing survival means an increasing population, but for the coming decades it also implies an increase in the number of the oldest old people, that is, those older than 80 and 90. In 2015, Europe had the most aged population of older persons, with people aged 80 years or over accounting for nearly one in five of those aged 60 years or over in the region. According to projections, the proportion of the population aged 80 years or over will surpass 25% by 2040, and by 2050 the oldest old people are projected to account for 29% of older persons in European countries. This is not all, however, studying the oldest old population in Europe, Robine and Saito, 2009 reported that the number of centenarians had increased by a factor of 1.4 during the period between 1946 and 1956, by a factor of 1.7 between the period 1956 and 1966 and by a factor of 1.9 during the four decades starting from 1966. They concluded that this development “demonstrates that centenarians were not exceptional people […]. There were at least 235 male and 1,098 female centenarians in the 14 countries of this group” (see Figure 2). Worldwide, the number of centenarians in 2010 was 350,000 and projections made by the United Nations Department of Economic and Social Affairs (2016) estimate that this will be 10 times higher by 2050, rising to 3,676,000.

In sum, we are rapidly approaching a new and fascinating world in which more older-old cohorts are increasingly aging our society. Given the rectangularization of the survival curve and the compression of morbimortality with the postponement of illness and disability, we must consider that the oldest old or the very old imply a threat both for the individual and society because the probability of illness and disability continues to be associated with age. The biomedical approach is without a doubt effective in improving this situation by applying innovative biotechnology, which furthers an intense debate arising from the possibility of the postponement of aging while also promoting public enthusiasm. This new world could be considered a challenge for society and for the individual which requires all of our problem-solving skills to achieve becoming older healthily, actively, productively, and with vitality. It is true that in recent decades, a new paradigm of successful aging (for a review, see Fernández-Ballesteros et al., 2019) has been developed, but very little is known about the relative contribution of different life conditions, such as biological, socio-economic, environmental factors, etc.

This article, as a prolongation of the discurse by Fernández-Ballesteros (2017) to the Spanish Academy of Psychology, aims to present the state of the art regarding factors accounting for aging and longevity, focusing on psycho-behavioral (PB) factors. The next section is focused on the polar classification “Intrinsic Versus Extrinsic Determinants of Aging,” highlighting several conditions that must be considered. The section on “The Interacting Process of Aging” describes the absence of research on the extent to which bio-psycho-behavioral and socio-cultural conditions contribute to the variance in longevity or healthy survival. Authors of longitudinal, multicohort and family studies conclude that never before have so many people reached older ages with better health, leading to the emergence of “The new paradigm of healthy, successful, active, productive aging” or “aging well.” In this section the authors examine this new paradigm and the relationship between the relative accounted variance for longevity and/or “positive” longevity, and psycho-behavioral intervention factors. The final section, “Psycho-Behavioral Factors Associated With Health and Survival” introduces the importance of psycho-behavioral factors influencing health and survival. This section also contains the “Concluding Remarks.”

## Intrinsic Versus Extrinsic Determinants of Aging

Bio-demographers, biologists, sociologists, gerontologists and other experts on aging attribute 20 to 25% of longevity to genetic components and 75 to 80% to environmental factors. Two main flaws emerge regarding this issue: (1) genetic and environmental factors are not independent but interactive factors; (2) the dynamic interrelations between these processes, taking into account the constant socio-historical, interindividual and intrapersonal changes across the life cycle depending on the life stage of a given society have been neglected, and finally, (3) environmental conditions are a hotchpotch which includes physical, economic, social, and cultural aspects alongside *personal conditions*, which include both behavioral (such as lifestyles) but also psychological characteristics (see section “Psycho-Behavioral Factors Associated With Health And Survival”); the question must be raised whether any personal condition is an environmental circumstance, and with respect to whom, for example, the individual who carries these internal or genetic factors. Thus, while socio-environmental and personal conditions must be considered separately but also in interaction, their relative contribution remains unknown; and, perhaps most importantly, this issue has been neglected not only by the diverse disciplines involved but also by psychology and psychologists.

In spite of these flaws, two major constructs have been considered when trying to explain *why* some people live longer and more healthily than others: *genetic* or *intrinsic* components (i.e., biomedical) and *environmental* or *extrinsic* factors both have been the subject of research interest and investment in empirical studies. Let us only mention here, from an epigenetic standpoint, that this polar classification could lead to *misconceptions* in the field since *both factors are interdependent*, in other words, intrinsic or genetic factors are to some extent influenced by extrinsic or environmental conditions, and the latter could be partially explained by the former. Therefore, the attempt to quantify the contribution of these two types of factors – which are not mutually exclusive - to individual differences in aging, survival and longevity, without taking into consideration their mutual interactions, could be considered an epistemological and methodological flaw which must be recognized and critically examined.

There are many studies seeking to list the most important determinants, both *genetic* and *environmental*, posited as predictors of longevity in industrialized countries, and this distinction has continued until today. Let us describe only the European project GEHA (Skytthea et al., 2011), initiated with the aim of identifying genes involved in healthy aging and longevity (through 2,500 sibling pairs of 90 years of age). The GEHA project thus represents a unique source in the search for genes related to healthy aging and longevity. Several interesting results arose from this project, such as those found by Cevenini et al. (2014): absence of cognitive impairment and physical disability, high hand grip strength scores and body mass index (BMI) values, “excellent/good” self-reported health, high hemoglobin and total cholesterol levels and low creatinine levels; this, then, is the first precursor of our project.

In a step forward from these genetic studies, and considering influences on longevity in interaction with lifestyle (behavioral) factors, compelling results are reported by Lindahl-Jacobsen and Christensen (2019), who conclude that “*the increasing evidence that the environment interacts with genes to alter their causal effects makes an integration of the environmental factors in the exploration for genes associated with longevity a key component in order to understand the mechanisms of aging*”(p 10). It is important to underline that this is an exception; there are very few studies taking PB factors into account from a bio-demography perspective which analyze data regarding the relative contribution of G and E, including PB factors of longevity and survival as well as the interactions among all of them along the life span. It seems that this could be a result of the older biomedical sciences, so well-established in the field of aging, neglecting relevant impulses from the younger psychology.

Moreover, as mentioned previously, individual differences in psychological factors (personality and intelligence characteristics) are accounted for by genetics; thus, as Vaupel et al. (1998) stated, “20 to 25% of the variation in *adult life spans* can be attributed to genetic variation among individuals; heritability of life span is also modest for a variety of other species. The possibility that polymorphisms may play an increasing role with age is supported by evidence of increases with age in the genetic component of variation in *both cognitive and physical ability*” (Vaupel et al., 1998 p. 859, italics added). This hypothesis has not yet been tested but is supported by McGue et al. (2014), who have suggested that the most powerful design for investigating the nature of associations between psychology and aging outcomes is a twin study (monozygotic –MZ- and dizygotic –DZ), and also recommend the use of available database sets of both longitudinal studies of aging and active aging promotion evaluation studies.

In sum, it could be posited that since psychological and behavioral factors have genetic as well as environmental interactive bases, research on this topic (1) must take into consideration the variance accounted for by interactions between PB, genetic, and environmental conditions for longevity and attempt to disentangle these interactions at different life-span stages; (2) must overcome the reductionist categorization of intrinsic/genetic vs. extrinsic/environmental factors introducing psycho-behavioral conditions as personal circumstances linking testing new comprehensive models through the results yielded; and finally, (3) must incorporate quantitative and qualitative results of longitudinal studies of twins and aging as well as cross-sectional, cohort, and experimental intervention research. Databases are now readily available, and big data allows the most sophisticated analysis; hence the interdisciplinary scientific community, including psychologists, are called to contribute to this enterprise.

## The Interacting Process of Aging

A human being is a bio-psycho-cultural entity, an active agent constructing him or herself across the life span in interaction with an active world, and across an ongoing and dynamic process (Staats, 1975; Gould, 1981; Bandura, 1986). Intraindividual and interindividual differences usually attributed to age are not exclusively due to such but also to the ongoing and dynamic process through which the individual -as a biological organism and his or her behavioral and psychological conditions- interact with external factors such as socio-cultural, economic, environmental, etc. During the process of aging, what the human being does, thinks and feels, and how he or she interacts with the environmental and his or her historical circumstances are decisive for aging outcomes.

As Birren (1996) pointed out, the science of gerontology is mainly devoted to the multidisciplinary study of aging, age and the aged; therefore, even the scientific subject of gerontology embraces primary (due to age) and secondary (due to diseases) aging and their individual differences as well as the process of aging itself and aged people. This diversity in the subject of study has influenced a certain bias in the selected topics: authors studying the “aging” process emphasize rather small intraindividual changes; authors studying differences between “age” groups aim to focus on interindividual differences attributed to age; and finally, those authors studying “the aged” are devoted to highlighting illnesses, impairments, and needs of care, thus focusing on impairment and suffering during the process of aging. Birren also emphasizes multidisciplinarity as the most relevant characteristic of the study of aging and, therefore of gerontology, that is, the requirement that aging, age, and the aged must be studied from a bio-psycho-social perspective and cannot be reduced to just one of the disciplines involved. Therefore, it may be concluded that this biomedical, psychological or social reductionism cannot be adopted by the entire field of study.

On the other hand, the issue not only refers to the specific view from different theoretical perspectives but also from the point of view of the subject of knowledge: *the dynamic process of aging.* Thus, as Gould (1981) emphasized, psycho-social functioning cannot be considered under the same principles as those guiding the study of organisms as biological entities; human functioning is also determined by socio-cultural context. Nevertheless, following Gottlieb and epigenetic theory, genetic and neural activity interacts with the environment and behavioral conditions along the same lines suggested by Bandura (1986) in his socio-cognitive theory and developed by Staats (1975) in his paradigmatic behaviorism model, as shown in Figures 2A,B.

Supporting the first figure, for example, Lindahl-Jacobsen and Christensen (2019) tested the importance of the life cycle on the effects of genes (e.g., APOE) and lifestyles depending on the particular period of the life cycle, as has been noted. This is also in agreement with Bandura (1986), who posited from his socio-cognitive theory that psychological functioning is determined by the reciprocal interactions between the person (including his/her biological conditions), his/her behaviors, and the socio-cultural context. Finally, it is also in agreement with Staats (1975), who in his paradigmatic behaviorist theory posits that a given present behavior can be explained through a life-long process in which historical environmental conditions (E1) interacting with the organism (O1) in its biological sense generate a Basic Behavioral Repertoire (BBR); these BBRs further interact with current environmental conditions (E2) and with current biological circumstances (O2 and O3), thereby determining present behavior. These relationships can vary at different stages of the life course, thus constituting a vital and unique equation reflecting the fact that at any given time individuals are trying to cope and give adaptive responses to a variety of life circumstances. For example, during childhood and adolescence, family socio-economic position is highly important for development (cognitive-languages, sensorimotor or emotional-motivational behavioral repertoires), while during adulthood IQ, personality, preferences, coping styles, etc., are going to be much more important than social and economic family circumstances. Thus, as Bandura, Staats, and other interactionist authors have pointed out, the person (and his/her biological characteristics and BBR) interacts with his/her socio-cultural conditions, and the individual’s behaviors can even influence organismic and environmental circumstances.

In sum, the mere concept of “age,” the process of aging or the individual differences in how a given person in a given society ages are bio-psycho-socio phenomena which take into consideration the interactive nature of those conditions. The process of aging cannot be reduced to biomedical conditions but neither can it be reduced to socio-cultural or behavioral ones. In order to examine whether bio, psycho-behavioral, socio-cultural conditions contribute to the variance in longevity or healthy survival, their interaction across the life span must be taken into consideration; nevertheless, this prerequisite is currently almost completely absent in research attempting to account for longevity and survival, reminding us of the André Gide quote: “*Everything has been said before, but since nobody listens we have to keep going back and beginning all over again*.”

## The New Paradigm of Healthy, Successful, Active, Productive Aging or “Aging Well”

As a result of human and social development -including biomedical and communication technology progress, mandatory education, universal health care, hygiene and other public policies- the human life span increased worldwide during the 19th and 20th centuries until the present, and in developed countries, life expectancy at birth has even doubled, increasing at a constant rate of 2 to 3 months per year, which suggests a new panorama: the unknown ceiling for human life (Vaupel, 1997). Also, from the third part of the 20th century onward, the fertility rate has been declining all over the world down to the replacement level in most developed countries. These two demographic changes have produced a large increase of older people both in absolute and relative numbers worldwide, and this can be considered the antecedent of the *aging of aging*: at the end of the 20th century it is the oldest old (those older than 80–90) which is the most rapidly increasing age group. Therefore, the aging of the population as well as the aging of aging can be considered as one of the most important demographic revolutions in human history.

Since science is *accumulative* and *historical*, new findings regarding a subject under scientific study can change its conceptualization. From an individual point of view, a 70-year old man or woman born in the first third of the 20th century with a life expectancy at birth of about 50 years today not only has a high probability of living longer than his or her parents did but also of living longer in better bio-psycho-social conditions. Data from all the disciplines converging in gerontology, such as the results of longitudinal, multicohort, and family studies (see, Vaupel et al., 1998; Schaie, 2005a, b; Christensen et al., 2009) spur authors to conclude that never before have so many people reached older ages in better health. In sum, these changes from a demographic and individual point of view support the existence of a new perspective in the study of aging, age and the aged.

From an evidence-based point of view, it was during the last decades of the 20th century when the so called “new paradigm” or “revolution” started in the field of aging research and in a broad sense in the science of gerontology; this was a positive view. Pioneers in this new paradigm are authors from several gerontological disciplines, that is, from the fields of biomedicine and social sciences, such as Fries and Crapo (1981), Rowe and Kahn (1987), Fries (1989), or Baltes and Baltes (1990).

The new paradigm is based on three main theoretical assumptions: (1) the broad *variabilit*y of the forms of aging tested in most parameters of aging (e.g., Baltes and Smith, 2003) (2) the *plasticity* of the human organism as well as its reserve capacity (e.g., Stern, 2002); (3) the *selection, optimization and compensation* mechanisms of aging across the life span, as described by Baltes and Baltes (1990), and finally (4) empirical proof regarding the *postponement of aging* during the last century as well as the *morbimortality compression* already described above in the demographic section. All these examples of progress allow the emergence and development of this new, positive model of aging, taking into consideration the diversity of verbal labels with a very similar empirical reference (see Peel et al., 2005). This paradigm is in fact based on scientific data regarding health, longevity, and aging well but not on speculation about the possibility of developing a transgenic human nature, the death of death, futurism, or transhumanism, perspectives mostly proposed by the Singularity University.

The new paradigm described here is postulated after the observation of several ways of aging synthesized in “usual.” “pathological,” and “successful” aging as posited by Rowe and Kahn (1987), discussed by, among others Fries and Crapo (1981), Rowe and Kahn (1987), Baltes and Baltes (1990), Fernández-Ballesteros et al. (2019), and labeled by WHO (2002/2012) as “active aging.” This re-evolution was modified, however, by the WHO (2015/2018) when the *active aging* conceptualization was abandoned in a backward step by reverting to “*healthy*” *aging*, thereby reducing it to functionality, and worse, eliminating any reference to psychosocial concepts and going back to the search for intrinsic determinants.

Parallel to the emergence of this new paradigm, in order to measure the length of healthy/unhealthy or functional/dysfunctional life expectancy, new demographic indicators have been developed (based on several data sources: self-reports, biomedical data, disability surveys), among them disability-free life expectancy (DFLE) and healthy life expectancy (HLE). After examining HLE in developed and developing countries WHO (2002/2012), it can be estimated that the lowest unhealthy life expectancy is about 7.3 years (Switzerland, LE = 79.8) and the highest is 15.3 (Peru, LE = 72.4). In sum, country differences in the prevalence of healthy or disability-free life expectancy are very high, likely explained by environmental or extrinsic population factors (behavioral or environmental factors); moreover, these new population indexes which have been developed do not embrace longevity, and therefore we cannot answer the question regarding the extent to which PB factors can account both for longevity and successful longevity.

**FIGURE 3:**
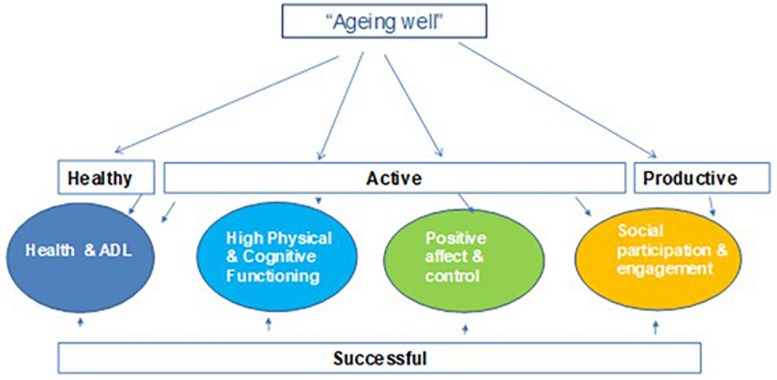
Four domains model of aging well (modified from Fernández-Ballesteros, 2019). Reprinted with the permission of the copyright holder. Copyright by Cambridge University Press.

This positive view of aging has adopted several verbal labels: “healthy” (WHO, 1997), “successful” (Rowe and Kahn, 1987; Baltes and Baltes, 1990), “optimal” (Palmore, 1999), “vital” (Erikson et al., 1986), “productive” (Butler and Gleason, 1985), “active” (WHO, 2002/2012), “positive” (Gergen and Gergen, 2001) or, simply “aging well” (Fries, 1989) or “good life” (Bearon, 1996). It is important to emphasize that all these terms are used almost interchangeably by experts (e.g., Peel et al., 2005; Depp and Jeste, 2006). Nevertheless, recently, Fernández-Ballesteros (2019) has tried to distinguish those more frequently used technical verbal terms (healthy, successful, active, and productive aging) based on the four domains most commonly cited in authors’ definitions: *Health and Activities of Daily Living (ADL); High physical and cognitive functioning; Positive Affect and Control*, and *Social Participation and Engagement.* Thus, as shown in Figure 3, these “related terms” can be characterized or reduced to one or several definitional domains. *Healthy aging*, for example, could be defined by only one of these domains: health and functionality (WHO, 2015/2018), along the same lines as *productive aging* is reduced to social participation and involvement (Butler and Gleason, 1985). Furthermore, *successful aging is* defined by three domains using Rowe and Khan’s definition (health and functioning, physical and cognitive competences, and social participation and engagement (not including positive affect and control) or by the four domains, if affect and control are included, taking the definition of successful aging by Baltes and Baltes (1990) or Carstensen et al. (2011), or *active aging* as defined by most of the authors in the field (Fernández-Ballesteros, 2019, p. 17).

It must be emphasized that this four-factor model was tested with a confirmatory factor analysis using two databases from two studies (several samples, several variables, assessed through several methods), independently yielding this psycho-behavioral four-domain model: health and functionality, physical and cognitive fitness, affect and control and social participation and engagement (Fernández-Ballesteros et al., 2013; Figure 3).

Furthermore, this four-domain model has been supported during recent decades by experimental data coming from psycho-social intervention programs. Thus, in order to promote and increase active or successful aging, several intervention programs have been developed, most of which have been tested by researchers and academics, while political efforts at different levels have also been planned by national and international organizations. Caprara and Mendoza (2019) have recently reviewed the programs which have been evaluated so far and emphasized that those which were more multidimensional and took psycho-behavioral actions into account yielded better results.

In sum, outcomes evaluation of successful aging programs did not include survival or “positive survival,” and there are no results regarding the relative accounted variance for longevity and/or “positive” longevity due to psycho-behavioral (so-called “environmental”) intervention factors. Moreover, very little research in twins, longitudinal, cross-sectional, cohorts, family or intervention studies about aging and outcomes research on successful aging has provided such information. Consequently, let us introduce only positive results regarding the importance of PB factors influencing health and survival which can support our basic assumptions here.

## Psycho-Behavioral Factors Associated With Health and Survival

Although our research group and the laboratory are devoted to the field of aging and psychology, during the last 30 years most of our research projects have been devoted to studying the effects of age on behavior and psychological functioning (see e.g., Fernández-Ballesteros et al., 2004). In doing so, we have reached the hypothesis that psycho-behavioral characteristics have a causal value across individual life spans similar to the way that interactions throughout human history between human behavior and psychological attributes are associated with human, social, and economic development. This is a good example of the need to consider such interactions with other bio-environmental and psychological factors (e.g., Fernández-Ballesteros and De Juan-Espinosa, 2000) and their potential influence on life expectancy and longevity during the 20th century.

### Intelligence

**FIGURE 4:**
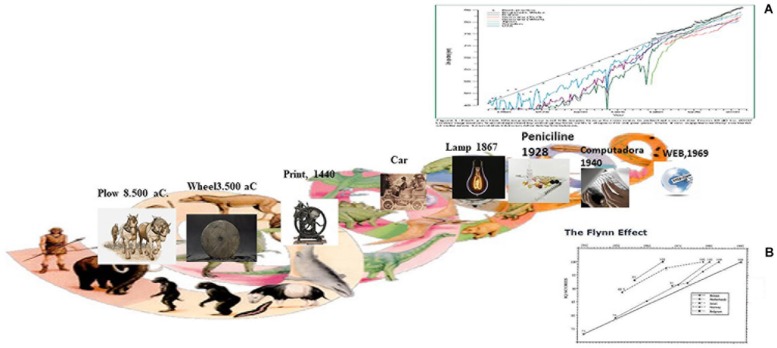
Products of human mind across history and, since 1850 increasing longevity and cognitive functioning. Reprinted with the permission of the copyright holders. **(A)** Reprinted from The Lancet, 374(9696), Christensen et al. (2009) Ageing populations: the challenges ahead, 1196–1208, 2009. With permission from Elesevier. **(B)** Reprinted with the permission of the copyright holder. Reprinted from American Psychological Association, Neisser, U. (Ed.). (1998). The rising curve: Long-term gains in IQ and related measures.

Starting with one of the most important psychological characteristics, human intelligence (mental abilities, competences, cognitive functioning), Figure 4 shows some inventions of the human mind across history as well as the evolution of longevity, starting in 1850 to date, and parallel Figures show the increase in measures of intelligence.

This combined illustration shows some products of the human mind throughout history: the plow (8500 BC), the wheel (3500 BC), the printing press (1440), the car (1885), the lamp (1897), antibiotics (1928), the computer (1940), the web (1960), the smartphone (1992). All of these inventions could be considered products of human phylogenesis in the bio/psycho/socio/environmental interactions as well as of socio-economic and technological developments, and from a certain point onward of the increase in life expectancy. As we can see, life expectancy from 1850 to 2005 (see A) follows a very similar trend to that of the growth of IQ (1910–1990), the so-called Flynn effect, in several countries in the second half of the 20th century (1942–1992) (see B), as well as the continually growing data on reasoning, processing speed and spatial aptitudes from the Schaie (2005a) Seattle Longitudinal Study (cohort study), which also shows the effects of changes in education.

From this historical bird’s eye view, the following conclusions can be drawn: (1) In cross-sectional designs, an increase in the scores yielded by intelligence tests, known as the Flynn effect, has been observed in different countries and with different measurement instruments; (2) Since the beginning of longitudinal studies on aging, it has been highlighted that participants with lower scores in intellectual functioning die earlier than those with higher intelligence measures, and (3) Many primary mental abilities are improved, so that the youngest cohorts score higher on a series of mental aptitudes.

Although there were some pioneering studies, it is not until the last decade of the last century that publications began to highlight the importance of intelligence measures at an early age as risk factors and also predictors of mortality (O’Toole, 1990; O’Toole and Stankov, 1996; Snowdon et al., 1996, 1999). The association between intelligence and aptitudes with aging parameters have also been informed by the new approach regarding intelligence called *cognitive epidemiology* proposed by Lubinski and Humphreys in 1997 (see Deary, 2010) and is defined as the field of research that examines the associations between intelligence test scores and health, morbidity, and mortality (see: Gottfredson and Deary, 2004; Batty et al., 2007; Deary et al., 2009). This approach is the source of the following remarks and conclusions: (1) As is well known, the word “intelligence” comes from the Latin *inteligere* (composed of *intus*: “between” and *legere*: “choose”), so intelligence could be defined as describing *one who knows how to choose.* (2) It implies a general capacity, *the ability to reason, plan, solve problems, think abstractly, understand complex ideas, learn quickly, and learn from experience*. (3) In other words, the *intelligent person* is one who, in a given situation, *selects the most appropriate alternatives to provide a satisfactory solution*, and (4) It is a psychological variable that must have played an extraordinary role in the process of hominization and phylogenesis, and must have been involved in the developments over the last 100 years.

Cumulative data on intelligence tests and aptitudes in standardized testing are highly consistent and show an inverse association with all causes of risk of death in childhood and throughout adulthood; in other words, high scores on intellectual functioning seem to be protective against disease and mortality. A summary of the scientific confirmation has appeared in Scientific American (see Hambrick, 2015).

Calvin et al. (2011) carried out meta analyses of terms related to cognitive and intellectual skills (“Aptitude or Cognition” or “Cognitive function” or “Cognitive ability” or “Cognitive characteristics” or “Cognitive style” or “intellectual ability” or “Intelligence measures” or “Intelligence quotient” or “Intelligence test” or “Intelligence” or “IQ or Language test” or “Memory” or “Mental ability” or “Mental capacity” or “problem-solving” or “Problem solving” or “Psychological performance” or “Psychometrics”) in EMBASE, MEDLINE and PSYCHINFO databases, included 16 unrelated studies, comprising 22,453 deaths among 1,107,022 participants. Meta-analytic results showed that a 1-standard deviation (SD) advantage in cognitive test scores was associated with a 24% (95% confidence interval 23–25) lower risk of death, during a 17- to 69-year follow-up. There was little evidence of publication bias (Egger’s intercept = 0.10, *P* = 0.81), and the intelligence–mortality association was similar for men and women. Controlling for adult SES and for education attenuated the intelligence–mortality hazard ratios by 34 and 54%, respectively, so authors conclude that SES does not seem to influence the intelligence-mortality association.

Gottfredson (2004), Gottfredson and Deary (2004) and Deary (2009) have proposed three potential arguments to explain the association between intelligence, health and longevity: (1) It could represent an indicator of the organism’s general “integrity”; (2) It could be an essential basis for the educational and professional, and therefore socio-economic level; (3) It could be the basis for BBRs for healthy behaviors; and all of this with shared genetic background. Batty et al. (2007) propose a model of influences on pre-morbid IQ and potential pathways linking pre-morbid IQ with later mortality: the authors highlight four potential confounding variables: socioeconomic disadvantage, birth weight, parental intelligence, and somatic/psychiatric illness. And five mechanisms which may explain how higher intelligence in early life (measured by IQ) might be protective against premature mortality: through adult socioeconomic advantage, improved disease/injury prevention, better disease/injury management, reduced psychiatric disease, and “body system integrity.”

Let us move on to Emotions and affect as a second field in the study of psychology and health.

### Emotions and Affect

Among the emotions, the most important psychological construct is *negative affect* (anxiety, depression, hostility, and stress) (Watson and Clark, 1984; Hu and Gruber, 2008; Carstensen et al., 2011; Diehl et al., 2011; Ong et al., 2011; Hershfield et al., 2013), considered a risk factor of *disease and mortality*, which seems to pose a threat to physical health in addition to being the essential element in negative mental health in general. Coping resources and how individuals mobilize and use them along their life course, to face demands in everyday life, arises as key elements for health in later life. Stress has multiple effects on the body, autonomic nervous system, hormone levels, and brain activity (Cacioppo et al., 1998); and accumulation of stress affects health by increasing the risk of disorders (Schroder et al., 2011); Following Folkman et al. (1986) stress and coping explains about 50% of the variance in psychological symptoms.

But, conversely, in an exhaustive review, Pressman and Cohen (2005) point out that positive affect reduces the probability of mortality and is a protective factor against cerebrovascular accidents and the common flu, and positive emotions are further associated with improvements in cardiovascular, endocrine and neuroimmune systems. Ostir et al. (2004) make the point that positive affect postpones fragility by 7 years. In sum, there is strong empirical support that *positive affect* reduces the probability of mortality in older people. Similarly, well-being, in both healthy and pathological populations, exerts a protective role, as reported in a meta-analysis by Chida and Steptoe (2008).

An important hypothesis by Fredrickson and Levenson (1998), “broaden-and-build,” is that positive affect can be an amplifier of the individual’s resources, which broadens the meaning of positive emotions, spreading out their role across other psychological factors.

Considering that the broad majority of research on affect and health comes from “the first-world,” the question arises as to whether these relations are context dependent, whether the relationship between affect and health is therefore specific to developed countries and not relevant to other contexts. Pressman et al. (2013) question whether the connection between emotion and health persists in the different regions of the world. To clarify the question, the authors analyzed 142 countries (*N* = 150,048) (52.1% female; mean age: 39.39; SD = 16.9; age range = 15–99 years) which participated in the first wave of the Gallup World Poll, an annual survey of approximately 1,000 individuals from more than 142 countries which provides a representative sample of 95% of the world’s population (information about the development of the survey, ethical considerations, and survey procedures including items and self-reports used can be obtained online at www.gallup.com/strategicconsulting/worldpoll.aspx). The results showed that both positive and negative emotions exhibited unique, moderate effects on self-reported health, and together, they accounted for 46.1% of the variance. These associations were stronger than the relative impact of hunger, homelessness, and threats to safety. Furthermore, connections between positive emotion and health were stronger in low-GDP countries than in high-GDP countries. In sum, emotion matters for health around the world.

In the emotion-motivation field, *personal control* reflects individual’s beliefs regarding the extent to which they are able to control or influence outcomes. Psychological theory and research suggest that personal control beliefs strongly predict future behavior, health, and illness. Positive attitudes and beliefs of control seem to influence the kind of behaviors necessary for adherence to stimulating mental activities and healthy behaviors for cognitive aging (e.g., adherence to physician regimens and exercise programs, being active in everyday life). Moreover, high self-efficacy, the individual’s belief that he or she is capable of achieving a desired goal in a particular situation (e.g., Bandura, 1978) and high personal control, the individual’s beliefs about his or her control or influence in the outcomes (Skinner, 1996), strongly influence engagement in behaviors with effects on everyday functioning (Hertzog et al., 2008).

From the psychosocial perspective of role theory (Krause and Shaw, 2000), perceptions of control – over one’s life or over specific roles – are said to have an effect on mortality. These authors carried out a field study with a representative sample of older adults (*N* = 884) and concluded that, although the perception of control over one’s life reduces mortality, control over specific roles does not seem to have an effect on this important parameter of health.

Continuing along the same line of research, Levy et al. (2002a) re-examined OLSAR (Ohio Longitudinal Study of Aging and Retirement), taking *Attitudes about own aging* at baseline, when participants were about 50 years old. An analysis of mortality when the study finished revealed that those participants reporting a positive view about their aging process lived 7.5 years longer than those with negative attitudes, while also enjoying better health for the duration of the study (Levy et al., 2002b). Other motivational aspects such as resilience or achievement motivation also yielded promising results.

### Personality

Personality is without doubt the broadest psychological domain but, briefly, it refers to individual differences in characteristic patterns of thinking, feeling, and behaving.

One of the first studies to emerge from longitudinal archival data was Terman’s study of genius and gifted children (Terman and Oden, 1947), which analyzed 856 boys and 672 girls (mean age 11 years). By 1991, 50% men and 35% women were dead (50% approximately). At this point, Friedman et al. (1995) collected information from mortality records and carried out a survival analysis to predict longevity and cause of death. Psychosocial life events such as parents’ divorce seem to be related to mortality (35% more than those that did not go through such an event). Among the psychosocial factors, conscientiousness can be considered a protective factor against mortality by cardiovascular disorders and cancer, and this protection remains after controlling for the consumption of alcohol and tobacco and other personality factors and, in second place, unhealthy habits like alcohol and tobacco are shown to be predictors of mortality. Kern et al. (2009) show similar findings to those of Friedman et al. highlighting the protective role of conscientiousness against mortality.

In brief, research on some personality traits have yielded high associations with health and longevity; for example, *extraversion, stability, affability*, *tenacity*, and *openness to experience* predict survival from any cause of death (Wilson et al., 2004, 2005), while *low tenacity, low persistence*, *poor self-control*, and *low long-term planning capacity* are associated with a high risk of mortality (e.g., Wilson et al., 2004). These associations remain after adjustment for healthy behaviors, marital status and education, as corroborated by other meta-analytical studies (Jokela et al., 2013). Finally, continuing this line of research, personality traits have been found to mediate the appraisal of stress: neuroticism has a strong association to stress reactions (e.g., Newbury-Birch and Kamali, 2001; Deary et al., 2003), on contrary, people high on the trait extraversion may suffer less under stress and experience more positive affect (Watson and Clark, 1992).

To date, studies have focused on older adults, but an inevitable question arises: is the predictive capacity of mortality the same from early childhood to adult life or different? Martin et al. (2007) tried to answer this question by carrying out a prospective longitudinal cohort study of 1,253 male and female over 7 decades (1930–2000). The findings, including an additional 14-year follow-up in old age, revealed that levels of conscientiousness, measured independently in childhood and adulthood, predicted mortality risk across the full life span. Conscientiousness was the only personality trait which allowed mortality risk to be predicted from 1950 to 2000. Neuroticism, sociability or extraversion did not significantly predict mortality in those five decades. Finally, these authors highlight the importance of these personality traits, associated with lifestyles and risky behaviors, which emerge as factors strongly associated with health, endorsing studies by Friedman et al. (1995) and Kern et al. (2009).

### Psychosocial Functioning

Among psychological domains, psychosocial functioning may play a role, constituting the social expression of certain personal aspects such as social abilities, assertiveness and other psychological attributes close to personality, alongside others such as social networks and social supports, which are closer to external/environmental conditions. Social ties are not only essential for psychological well-being in old age but also have a role in longevity and health; a socially engaged lifestyle is associated with positive results (Bosma et al., 2002; Zunzunegui et al., 2003; Barnes et al., 2004). A set of studies have examined the predictive power of social networks for *survival*. After adjusting for age, sex, race and baseline health status, people with a high “social network index” had a 50% lower risk of mortality than people with the lowest (Mendes de Leon et al., 2003). The association between social relationships and the prevalence, incidence and recovery from disability has been well established. Results have yielded a robust cross-sectional association between social engagement and disability; more socially active persons reported lower levels of disability than their less active counterparts. Results also showed that the protective effects of social engagement diminish slowly over time (Zunzunegui et al., 2005).

### Healthy Lifestyles

Finally, following the determinants of active and healthy aging posited by the WHO (2002/2012), there is an important corpus of empirical evidence regarding the association between health and psychological domains, among them so-called healthy lifestyles which, as already mentioned, are considered to be environmental/extrinsic determinants of survival and longevity. These lifestyles are, in fact, behavioral repertoires learnt across the life span based on individual psychological functioning: regular physical exercise, healthy diet, no smoking and drinking moderate amounts of alcohol are the lifestyles with the greatest amount of supporting empirical evidence from meta-analyses (e.g., Ford et al., 2012; Loef and Walach, 2012) and recognized by international organizations (WHO, 2002/2012, 2015/2018). In this section, we are not referring to these behavioral items but only to the psychological factors with strong relationships with health in the field of intelligence, personality, affect and motivation, and psychosocial relationships.

In this respect, it is important to highlight that adherence to behavioral routines and/or interventions regarding health and promotion of healthy life styles is mediated by psychological and intellectual factors: the understanding of health literacy, self-efficacy or conscientiousness, as well as networks of social support (for a review see Berkman, 1995).

Regarding health literacy, Baker et al. (2007) highlight that insufficient capacity to understand health literacy predicts all mortality causes of older adults living in community, and even point out that fluency in health reading literacy is more powerful than education. Bostock and Steptoe (2012) show similar findings in a longitudinal study of 7,857 older adults (mean age 52 years) from the English Longitudinal Study of Aging. After adjusting for personal characteristics, SES, baseline health, and health behaviors, low understanding of health literacy is seen to have a high-level effect in all mortality causes, as does the influence of intellectual abilities on the comprehension capacity of health literacy.

Regarding conscientiousness and its involvement in healthy lifestyles and behaviors, and in maintaining health-related behaviors and treatment, Bogg and Roberts (2004) conducted a meta-analysis of conscientiousness-related traits and the leading behavioral contributors to mortality in the United States. The results show that conscientiousness-related traits were negatively related to all risky health-related behaviors and positively related to all beneficial health-related behaviors.

### Physical Functioning

A final psycho-behavioral attribute with high relevance as predictive factor of longevity, mortality, and survival, while also predicting healthy and active aging, is physical functioning; most healthy-aging studies introduce physical conditions such as anthropometric, bio-behavioral measures (grip strength, speed, lung flow, step speed, balance, movement speed, etc.) as well as physiological records (heart rate, blood pressure, allostatic load, etc.) and molar psychological constructs such as vitality and the corresponding subjective appraisal of these physical conditions (e.g., Fernández-Ballesteros et al., 2003). Furthermore, higher BMI at ages 70 to 79 (Gustafson et al., 2003) and not being physically active in midlife may increase the risk of dementia and AD later in life (Kivipelto et al., 2005). Let us finally remember the strong association between cognitive and physical functioning and the hypothesis posited by Vaupel et al. (1998, p.859) related to the potential polymorphism variation in both cognitive and physical ability.

## Concluding Remarks

In response to the question regarding which factors account for aging and longevity, two major constructs have been considered from a bio-medical perspective and practice in trying to explain *why* some people live longer and more healthily than others: both *genetic* or *intrinsic* components (i.e., biomedical) and *environmental* or *extrinsic* factors have been the focus of research interest and investment in empirical studies. Two important flaws emerge from this classification and must be overcome: (1) From an epigenetic standpoint, this polar classification could lead to *misconceptions* in the field since *both factors are interdependent*, in other words, intrinsic or genetic factors are to some extent influenced by extrinsic or environmental conditions, and the latter could be partially explained by the former. (2) There are no specifications regarding the “environmental” factors which account for longevity, including physical, economic, social, and cultural aspects alongside *personal conditions*; both are behavioral (such as lifestyles) but also psychological characteristics. Thus, socio-environmental and personal conditions need to be considered separately as well as in interaction; the relative importance of their contribution remains unknown and, perhaps most significantly, this issue has been neglected not only by the diversity of disciplines involved but also by psychology and psychologists and therefore remains a pending issue.

In considering the successful or active aging paradigm, we have intervention evidence based on the importance of PB factors in the ways of aging as well as a broad corpus of research literature which supports our present hypothesis: cognitive functioning, emotion and control, personality, psychosocial, and physical conditions account to some extent for longevity and survival. In an aging world, however, much more interdisciplinary research must be conducted, and psychology and psychologists should be involved in the enterprise to determine the extent to which PB factors account for longevity.

In summary, a central question emerges regarding the lack of research into the effects of behavioral and psychological factors on longevity; this lack exists despite the fact that lifestyles (i.e., healthy behavioral factors) as well as other cognitive, motivational and emotional, and personality factors have been postulated as playing an important role for health, longevity, and survival, and despite the existence of strong evidence to this effect. The most likely answer could be related to a lack of multidisciplinarity (or better interdisciplinarity) as well as the importance of epigenetic aging.

## Author Contributions

RF-B: general idea and outline, writing, and manuscript review. MS-I: literature review, writing, and manuscript review.

## Conflict of Interest

The authors declare that the research was conducted in the absence of any commercial or financial relationships that could be construed as a potential conflict of interest.

## References

[B1] Baker D. W. Wolf M. S. Feinglass J. Thompson J. A. Gazmararian J. A. Huang J. (2007). Health literacy and mortality among elderly persons. *Arch. Intern. Med.* 167 1503–1509. 10.1001/archinte.167.14.1503 17646604

[B2] Baltes P. B. Baltes M. M. (1990). “Psychological perspectives on successful aging: the model of selective optimization with compensation,” in *Successful Aging: Perspectives From the Behavioural Sciences*, eds Baltes P. B. Baltes M. M. (Cambridge: Cambridge University Press).

[B3] Baltes P. B. Smith P. (2003). New frontiers in the future of aging: from successful aging of the young old to the dilemmas of the fourth age. *Gerontology* 49 123–135. 10.1159/000067946 12574672

[B4] Bandura A. (1978). The self system in reciprocal determinism. *Am. Psychol.* 33 344–358. 10.1037//0003-066x.33.4.344

[B5] Bandura A. (1986). *Social Foundation of Thoughts and Actions.* Englewood Cliffs, CA: Prentice Hall.

[B6] Barnes L. L. Mendes de Leon C. F. Wilson R. S. Bienias J. L. Evans D. A. (2004). Social resources and cognitive decline in a population of older african americans and whites. *Neurology* 63 2322–2326. 10.1212/01.wnl.0000147473.04043.b3 15623694

[B7] Batty G. D. Deary I. J. Gottfredson L. S. (2007). Premorbid (early life) IQ and later mortality risk: systematic review. *Ann. Epidemiol.* 17 278–288. 10.1016/j.annepidem.2006.07.010 17174570

[B8] Bearon L. B. (1996). Successful Aging: what does the “good life” look like? the forum for family and consumer issues. *North. California S. U.* 1 1–6.

[B9] Berkman L. F. (1995). The role of social relations in health promotion. *Psychos. Med.* 57 245–254. 10.1097/00006842-199505000-00006 7652125

[B10] Birren J. (ed.) (1996). *Encyclopedia of Gerontology. Aging, Age, and the Aged.* New York, NY: Pergamon Press.

[B11] Bogg T. Y. Roberts B. W. (2004). Conscientiousness and health-related behaviors: a meta-analysis of the leading behavioral contributors to mortality. *Psychol. Bull.* 130 887–919. 10.1037/0033-2909.130.6.887 15535742

[B12] Bosma H. van Boxtel M. P. Ponds R. W. Jelicic M. Houx P. Metsemakers J. (2002). Engaged lifestyle and cognitive function in middle and old-aged, non-demented persons: a reciprocal association? *Z Gerontol. Geriatr.* 35 575–581. 10.1007/s00391-002-0080-y 12491004

[B13] Bostock S. Y. Steptoe A. (2012). Association between low functional health literacy and mortality in older adults: longitudinal cohort study. *BMJ* 344:e1602. 10.1136/bmj.e1602 22422872 PMC3307807

[B14] Butler R. N. Gleason H. P. (1985). *Productive Aging: Enhancing Vitality in Later Life.* New York, NY: Springer Pub. Co.

[B15] Cacioppo J. T. Berntson G. G. Malarkey W. B. Kiecolt-Glaser J. K. Sheridan J. F. Poehlmann K. M. (1998). Autonomic, neuroendocrine, and immune responses to psychological stress: the reactivity hypothesis. *Ann. N. Y. Acad. Sci.* 840 664–673. 10.1111/j.1749-6632.1998.tb09605.x 9629293

[B16] Calvin C. M. Deary I. J. Fenton C. Roberts B. A. Der G. Leckenby N. (2011). Intelligence in youth and all-cause-mortality: systematic review with meta-analysis. *Int. J. Epidemiol.* 40 626–644. 10.1093/ije/dyq190 21037248 PMC3147066

[B17] Caprara M. G. Mendoza N. M. (2019). “Promoting successful aging,” in *Cambridge Handbook of Successful Aging*, eds Fernández-Ballesteros R. Robine J.M. Benethos A. (New York, NY: Cambridge Press).

[B18] Carstensen L. L. Turan B. Scheibe S. Ram N. Ersner-Hershfield H. Samanez-Larkin G. R. (2011). Emotional experience improves with age: evidence based on over 10 years of experience sampling. *Psychol. Aging* 26 21–33. 10.1037/a0021285 20973600 PMC3332527

[B19] Cevenini E. Cotichini R. Stazi M. A. Toccaceli V. Palmas M. G. Capri M. (2014). Health status and 6 years survival of 552 90+ Italian sib-ships recruited within the EU Project GEHA (GEnetics of Healthy Ageing). *Age* 36 949–966. 10.1007/s11357-013-9604-1 24323371 PMC4039258

[B20] Chida Y. Steptoe A. (2008). Positive psychological well-being and mortality: a quantitative review of prospective observational studies. *Psychos. Med.* 70 741–756. 10.1097/PSY.0b013e31818105ba 18725425

[B21] Christensen K. Doblhammer G. Rau R. Vaupel J. W. (2009). Ageing populations: the challenges ahead. *Lancet* 374 1196–1208. 10.1016/S0140-6736(09)61460-4 19801098 PMC2810516

[B22] Deary I. J. (ed.) (2009). Intelligence, health and death: the emerging field of cognitive epidemiology. *Intelligence* 37 517–634.

[B23] Deary I. J. (2010). Cognitive epidemiology: its rise, its current issues, and its challenges. *Pers. Individ. Dif.* 49 337–343. 10.1016/j.paid.2009.11.012

[B24] Deary I. J. Corley J. Gow A. J. Harris S. E. Houlihan L. M. Penke L. (2009). Age-associated cognitive decline. *Br. Med. Bull.* 92 135–152. 10.1093/bmb/ldp033 19776035

[B25] Deary I. J. Watson R. Hogston R. A. (2003). Longitudinal cohort study of burnout and attrition in nursing students. *J. Adv. Nurs.* 43 71–81. 10.1046/j.1365-2648.2003.02674.x 12801398

[B26] Depp C. A. Jeste D. V. (2006). Definitions and predictors of successful aging: a comprehensive review of larger quantitative studies. *Am. J. Geriatr. Psychiatry* 14 6–20. 10.1097/01.JGP.0000192501.03069.bc 16407577

[B27] Diehl M. Hay E. L. Berg K. M. (2011). The ratio between positive and negative affect and flourishing mental health across adulthood. *Aging Men. Health* 15 882–893. 10.1080/13607863.2011.569488 21562989 PMC3158962

[B28] Erikson E. H. Erikson J. M. Kivnick H. Q. (1986). *Vital Involvement in Old Age.* New York, ny: w. W. Norton & co.

[B29] Fernández-Ballesteros R. (2017). “Psicología y Envejecimiento: El comportamiento humano, ¿un factor causal de longevidad?,” in *Academia de Psicologia de España. Psicología para un mundo sostenible: Volumen I* (Madrid: Pirámide), 81–116.

[B30] Fernández-Ballesteros R. (2019). “Successful aging and related terms,” in *Cambridge Handbook of Successful Agin*, eds Fernández-Ballesteros E. R. Benetos A. Robine J. M. (New York, NY: Cambridge Press).

[B31] Fernández-Ballesteros R. Benethos A. Robine J. M. (eds). (2019). *Cambridge Handbook of Successful Aging*, New York, NY: Cambridge Press.

[B32] Fernández-Ballesteros R. De Juan-Espinosa M. (2000). “Socio-historical changes and intelligence gains,” in *Environmental Effects on Cognitive Abilities*, eds Sternberg R. J. Grigorenko Y. E. L. (Greenwich: JAI Press).

[B33] Fernández-Ballesteros R. Molina M. A. Schettini R. Santacreu M. (2013). “The semantic network of aging well,” in *Healthy Longevity. Annual Review of Gerontology and Geriatrics, 33, 2013*, eds Robine J. M. Jagger C. Crimmins E. M. (New York, NY: Springer).

[B34] Fernández-Ballesteros R. Staats W. (1992). Paradigmatic behavioral assessment, treatment, and evaluation: answering the crisis in behavioral assessment. *Adv. Behav. Res. Ther.* 14 1–29.

[B35] Fernández-Ballesteros R. Zamarrón M. D. Rudinger G. Schroots J. J. Hekkinnen E. Drusini A. (2004). Assessing competence: the european survey on aging protocol (ESAP). *Gerontology* 50 330–347. 10.1159/000079132 15331863

[B36] Fernández-Ballesteros R. Zamarrón M. D. Tárraga L. Moya R. Iñiguez J. (2003). Learning potential in healthy, mild cognitive impairment subjects and in alzheimer patients. *Eur. Psychol.* 8 148–160.

[B37] Folkman S. Lazarus R. S. Gruen R. J. Delongis A. (1986). Appraisal, coping, health status, and psychological symptoms. *J. Pers. Soc. Psychol.* 50 571–579. 10.1037//0022-3514.50.3.571 3701593

[B38] Ford S. F. Bergman M. M. Boeing H. Li C. Capewell S. (2012). Healthy lifestyle behaviors and all-cause mortality among adults in the United States. *Prev. Med.* 55 23–27. 10.1016/j.ypmed.2012.04.016 22564893 PMC4688898

[B39] Fredrickson B. L. Levenson R. W. (1998). Positive emotions speed recovery from the cardiovascular sequelae of negative emotions. *Cogn. Emot.* 12 191–220. 10.1080/026999398379718 21852890 PMC3156608

[B40] Friedman H. S. Tucker J. S. Schwartz J. E. Tomlinson-Keasey C. Martin L. R. Wingard D. L. Y. (1995). Psychosocial and behavioral predictors of longevity the aging and death of the “Termites”. *Am. Psycho.* 50 69–78. 10.1037//0003-066x.50.2.69 7879989

[B41] Fries J. Crapo L. M. (1981). *Vitality and Aging.* New York, NY: Freeman &Co.

[B42] Fries J. F. (1980). Aging, natural death, and the compression of morbidity. *New Engl. J. Med.* 303 130–135. 10.1056/NEJM198007173030304 7383070

[B43] Gergen M. M. Gergen K. J. (2001). Positive aging: new images for a new age. *Ageing Int.* 27 3–23. 10.1007/s12126-001-1013-6

[B44] Gottfredson L. S. (2004). Intelligence: is it the epidemiologists’ elusive “fundamental cause” of social class inequalities in health? *J. Personal. Soc. Psychol.* 86 174–199. 10.1037/0022-3514.86.1.174 14717635

[B45] Gottfredson L. S. Deary I. J. (2004). Intelligence predicts health and longevity, but why? *Curr. Direct. Psychol. Sci.* 13 1–4. 10.1111/j.0963-7214.2004.01301001.x

[B46] Gottlieb G. (1991). Experiential canalization of behavioral development: theory. *Dev. Psychol.* 27 4–13. 10.1037//0012-1649.27.1.4

[B47] Gould R. L. (1981). *The Mismeasure of Man.* New York, NY: Norton.

[B48] Gustafson D. Rothenberg E. Blennow K. Stehen B. Skoog I. (2003). An 18-year follow- up of overweight and risk of Alzheimer disease. *Arch. Intern. Med.* 163 1524–1528. 12860573 10.1001/archinte.163.13.1524

[B49] Hambrick D. Z. (2015). *Research Confirms a Link Between Intelligence and Life Expectancy.* Available at: http://www.scientificamerican.com/search/?q=Research+Confirms+a+ling+between+Intelligence&display=search&x=0&y=0 (accessed December 16, 2018).

[B50] Hershfield H. E. Scheibe S. Sims T. L. Carstensen L. L. (2013). When feeling bad can be good: mixed emotions benefit physical health across adulthood. *Soc. Psychol. Personal. Sci.* 4 54–61. 10.1177/1948550612444616 24032072 PMC3768126

[B51] Hertzog C. Kramer A. F. Wilson R. S. Y. Lindenberger U. (2008). Enrichment effects on adult cognitive development. *Psychol. Sci.* 9 1–65. 10.1111/j.1539-6053.2009.01034.x 26162004

[B52] Hu J. Gruber K. J. (2008). Positive and negative affect and health functioning indicators among older adults with chronic illnesses. *Issues Men. Health Nurs.* 29 895–911. 10.1080/01612840802182938 18649214

[B53] Jokela M. Batty D. G. Nyberg S. T. Virtanen M. Nabi H. Singh-Manoux A. Y. (2013). Personality and all-cause mortality: individual-participant meta-analysis of 3,947 deaths in 76,150 adults. *Am. J. Epidemiol.* 178 667–675. 10.1093/aje/kwt170 23911610 PMC3755650

[B54] Kern M. L. Friedman H. S. Martin L. R. Reynolds C. A. Y. Luong G. (2009). Conscientiousness, career success, and longevity: a lifespan analysis. *Ann. Behav. Med.* 37 154–163. 10.1007/s12160-009-9095-6 19455378 PMC2691806

[B55] Kivipelto M. Ngandu T. Fratiglioni L. Viitanen M. Kåreholt I. Winblad B. (2005). Obesity and vascular risk factors at midlife and the risk of dementia and alzheimer disease. *Arch. Neurol.* 62 1556–1560. 16216938 10.1001/archneur.62.10.1556

[B56] Krause N. Shaw B. A. (2000). Role-specific feelings of control and mortality. *Psychol. Aging* 15 617–626. 10.1037//0882-7974.15.4.617 11144321

[B57] Levy B. R. Slade M. D. Kasl S. V. (2002a). Longitudinal benefit of positive self-perceptions of aging on functional health. *J. Gerontol.* 57B 409–417. 10.1093/geronb/57.5.P409 12198099

[B58] Levy B. R. Slade M. D. Kunkel S. R. Kasl S. V. (2002b). Longevity increased by positive self-perceptions of aging. *J. Pers. Soc. Psychol.* 83 261–270. 10.1037//0022-3514.83.2.261 12150226

[B59] Lindahl-Jacobsen R. Christensen K. (2019). “Gene–Lifestyle Interactions,” in *Cambridge Handbook of Successful Aging*, eds Fernandez-Ballesteros R. Robine J. M. Benethos A. (New York, NY: Cambridge Press).

[B60] Loef M. Walach H. (2012). The combined effects of healthy lifestyle behaviors on all cause mortality. a systematic review and meta-analysis. *Preven. Med.* 55 163–170. 10.1016/j.ypmed.2012.06.017 22735042

[B61] Martin L. R. Friedman H. S. Y. Schwartz J. E. (2007). Personality and mortality risk across the life span: the importance of conscientiousness as a biopsychosocial attribute. *Health Psychol.* 26 428–438. 10.1037/0278-6133.26.4.428 17605562

[B62] McGue M. Skytthe A. Christensen K. (2014). The nature of behavioural correlates of healthy ageing: a twin study of lifestyle in mid to late life. *Int. J. Epidemiol.* 43 775–782. 10.1093/ije/dyt210 24711605 PMC4052129

[B63] Mendes de Leon C. F. Glass T. A. Berkman L.-F. (2003). Social engagement and disability in a community population of older adults. *Am. J. Epidemiol.* 157 633–642. 10.1093/aje/kwg028 12672683

[B64] Newbury-Birch D. Kamali F. (2001). Psychological stress, anxiety, depression, job satisfaction, and personality characteristics in preregistration house officers. *Postgrad Med. J.* 77 109–111. 10.1136/pmj.77.904.109 11161078 PMC1741911

[B65] Ong A. D. Mroczek D. K. Riffin C. (2011). The health significance of positive emotions in adulthood and later life. *Soc. Personal. Psychol. Compass* 5 538–551. 10.1111/j.1751-9004.2011.00370.x 21927620 PMC3173764

[B66] Ostir G. V. Ottenbacher K. Markides K. S. (2004). Onset of frailty in older adults and the protective role of positive emotion. *Psychol. Aging* 19 402–408. 10.1037/0882-7974.19.3.402 15382991

[B67] O’Toole B. I. (1990). Intelligence and behaviour and motor vehicle accident mortality. *Accid. Anal. Prev.* 222 11–221. 10.1016/0001-4575(90)90013-b 2393469

[B68] O’Toole B. I. Y. Stankov L. (1996). Ultimate validity of psychological tests. *Pers. Individ. Differ.* 136 99–716.

[B69] Palmore E. (1999). *Ageism: Negative and Positive.* New York, NY: Springer.

[B70] Peel N. M. McClure R. J. Bartlett H. P. (2005). Behavioral determinants of health ageing. *Am. J. Prev. Med.* 28 298–304. 10.1016/j.amepre.2004.12.002 15766620

[B71] Pressman S. D. Cohen S. (2005). Does positive affect influence health? *Psychol. Bull.* 131:925. 10.1037/0033-2909.131.6.925 16351329

[B72] Pressman S. D. Gallagher M. W. Y. Lopez S. J. (2013). Is the emotion-health connection a “first-world problem”? *Psychol. Sci.* 24 544–549. 10.1177/0956797612457382 23443305

[B73] Riley J. C. (2005). Estimates of regional and global life expectancy, 1800–2001. *Popul. Dev. Rev.* 31 537–543. 10.1111/j.1728-4457.2005.00083.x

[B74] Robine J. M. Saito. (2009). The number of Centenary in Europe. *Doc. Serv. Commun.* 21 569–598.

[B75] Roser M. (2018). *Life Expectancy.* Available at: https://ourworldindata.org/life-expectancy (accessed December 16, 2018).

[B76] Rowe J. W. Kahn R. L. (1987). Human aging: usual and successful. *Science* 237 143–149. 10.1126/science.3299702 3299702

[B77] Schaie K. W. (2005a). *Developmental Influences on Adult Intelligence: the Seattle Longitudinal Study.* New York, NY: Oxford University Press.

[B78] Schaie K. W. (2005b). What can we learn from longitudinal studies of adult development? *Res. Hum. Dev.* 2 133–158. 10.1207/s15427617rhd0203_4 16467912 PMC1350981

[B79] Schroder H. Reschke K. Gärtner A. Kaczmarek L. Sek H. Ziarko M. (2011). Psychosocial coping resources and health among Germans and Poles. *Pol. Psychol. Bull.* 42:114 10.2478/v10059-011-0016-8

[B80] Skinner E. A. (1996). A guide to constructs of control. *J. Pers. Soc. Psychol.* 71 549–570. 10.1037/0022-3514.71.3.549 8831161

[B81] Skytthea A. Valensinb S. Jeunea B. (2011). Design, recruitment, logistics, and data management of the GEHA (genetics of healthy ageing) project. *Exp. Gerontol.* 46 934–945. 10.1016/j.exger.2011.08.005 21871552 PMC3622890

[B82] Snowdon D. A. Greiner L. H. Kemper S. J. Nanayakkara N. Mortimer J. A. (1999). “Linguistic ability in early life and longevity: findings from the nun study,” in *The Paradoxes of Longevity. Research and Perspectives in Longevity*, eds Robine J. M. Forette B. Franceschi C. Allard M. (Berlin, Heidelberg: Springer), 103–113.

[B83] Snowdon D. A. Kemper S. J. Mortimer J. A. Greiner L. H. Wekstein D. R. Markesbery W. R. (1996). Linguistic ability in early life and cognitive function and Alzheimer’s disease in late life: findings from the nun study. *JAMA* 275 528–532. 8606473

[B84] Staats A. W. (1975). *Social Behaviorism.* Homewood, III: Dorsey Press.

[B85] Stern Y. (2002). What is cognitive reserve? theory and research application of the reserve concept. *J. Int. Neuropsychol. Soc.* 8 448–460. 11939702

[B86] Terman L. M. Oden M. H. (1947). *Genetic Studies of Genius: the Gifted Child Grows up.* Stanford, CA: Stanford University Press.

[B87] Vaupel J. W. (1997). The remarkable improvements in survival at older ages. *Phil. Trans. R. Soc. Lond. B* 352 1799–1804. 10.1098/rstb.1997.0164 9460063 PMC1692123

[B88] Vaupel W. Carey J. R. Christensen K. Johnson T. E. Yashin A. I. Holm N. V. (1998). Biodemographic trajectories of longevity. *Science* 280 855–860. 10.1126/science.280.5365.855 9599158

[B89] Watson D. Clark L. A. (1984). Negative affectivity: the disposition to experience negative aversive emotional states. *Psychol. Bull.* 96 465–490. 6393179

[B90] Watson D. Clark L. A. (1992). On traits and temperament: general and specific factors of emotional experience and their relation to the five-factor model: issues and applications. *J. Pers.* 60 441–476. 1635050 10.1111/j.1467-6494.1992.tb00980.x

[B91] WHO (1997). *WHOQOL: Measuring Quality of Life.* Geneva: World Health Organization.

[B92] WHO (2002/2012). *Active Ageing. A Policy Framework.* Geneva: World Health Organization.

[B93] WHO (2015/2018). *World Report on Ageing and Health.* Geneva: World Health Organization.

[B94] Wilson R. S. Krueger K. R. Gu L. Bienias J. L. Mendes, de Leon C. F. (2005). Neuroticism, extraversion, and mortality in a defined population of older persons. *Psychos. Med.* 67 841–845. 10.1097/01.psy.0000190615.20656.83 16314587

[B95] Wilson R. S. Mendes Cf Bienias J. L. Da. (2004). Personality and mortality in old age. *J. Gerontol.* 59B 110–116. 10.1093/geronb/59.3.p110 15118013

[B96] Zunzunegui M. V. Alvarado B. E. Del Ser T. Otero A. (2003). Social networks, social integration, and social engagement determine cognitive decline in community-dwelling Spanish older adults. *J. Gerontol.* 58B S93–S10. 12646598 10.1093/geronb/58.2.s93 PMC3833829

[B97] Zunzunegui M. V. Rodriguez-Laso A. Otero A. Pluijm S. M. F. Nikula S. Blumstein J. (2005). Disability and social ties: comparative findings of the CLESA study. *Eur. J. Ageing* 2 40–48. 10.1007/s10433-005-0021-x 28794715 PMC5547668

